# Association between oral antimalarial medication administration and mortality among patients with Ebola virus disease: a multisite cohort study

**DOI:** 10.1186/s12879-021-06811-3

**Published:** 2022-01-20

**Authors:** Logan Abel, Shiromi M. Perera, Derrick Yam, Stephanie Garbern, Stephen B. Kennedy, Moses Massaquoi, Foday Sahr, Dayan Woldemichael, Tao Liu, Adam C. Levine, Adam R. Aluisio

**Affiliations:** 1 Warren Alpert Medical School of Brown University, Providence, RI USA; 2 International Medical Corps, Washington, DC USA; 3 Center for Statistical Sciences, Department of Biostatistics, Brown University School of Public Health, Providence, RI USA; 4 Department of Emergency Medicine, Warren Alpert Medical School of Brown University, 55 Claverick Street, Room 274, Providence, RI 02903 USA; 5 Ministry of Health, Monrovia, Liberia; 6 College of Medicine and Allied Health Sciences, University of Sierra Leone, Freetown, Sierra Leone

**Keywords:** Ebola virus disease, Malaria, West Africa, Mortality

## Abstract

**Background:**

Empiric antimalarial treatment is a component of protocol-based management of Ebola virus disease (EVD), yet this approach has limited clinical evidence for patient-centered benefits.

**Methods:**

This retrospective cohort study evaluated the association between antimalarial treatment and mortality among patients with confirmed EVD. The data was collected from five International Medical Corps operated Ebola Treatment Units (ETUs) in Sierra Leone and Liberia from 2014 through 2015. The standardized protocol used for patient care included empiric oral treatment with combination artemether and lumefantrine, twice daily for three days; however, only a subset of patients received treatment due to resource variability. The outcome of interest was mortality, comparing patients treated with oral antimalarials within 48-h of admission to those not treated. Analysis was conducted with logistic regression to generate adjusted odds ratios (aORs). Multivariable analyses controlled for ETU country, malaria rapid diagnostic test result, age, EVD cycle threshold value, symptoms of bleeding, diarrhea, dysphagia and dyspnea, and additional standard clinical treatments.

**Results:**

Among the 424 cases analyzed, 376 (88.7%) received early oral antimalarials. Across all cases, mortality occurred in 57.5% (244). In comparing unadjusted mortality prevalence, early antimalarial treated cases yielded 55.1% mortality versus 77.1% mortality for those untreated (p = 0.005). Multivariable analysis demonstrated evidence of reduced aOR for mortality with early oral antimalarial treatment versus non-treatment (aOR = 0.34, 95% Confidence Interval: 0.12, 0.92, p = 0.039).

**Conclusion:**

Early oral antimalarial treatment in an EVD outbreak was associated with reduced mortality. Further study is warranted to investigate this association between early oral antimalarial treatment and mortality in EVD patients.

**Supplementary Information:**

The online version contains supplementary material available at 10.1186/s12879-021-06811-3.

## Introduction

The 2014–16 Ebola virus disease (EVD) epidemic in West Africa was the largest since the discovery of the virus in 1976 [1]. The mortality of EVD is high, averaging around 50%, although varies greatly, ranging from 25 to 90% across outbreak and treatment settings [1]. EVD is found in areas endemic to other infectious diseases and it can be difficult to differentiate EVD from other infectious diseases during initial evaluations [1]. Malaria, which is highly endemic in West Africa, can present with similar symptoms to EVD and was common among patients presenting to Ebola Treatment Units (ETUs), including among EVD-positive patients during the 2014–16 epidemic [1–7]. The World Health Organization (WHO) guidelines recommend empiric antimalarial treatment for patients with suspected EVD [8], and as such antimalarial medications are commonly given in EVD treatment protocols [2–4, 9–23].

Multiple antimalarial medications have been shown to have activity against the Ebola Virus (EBOV) in laboratory studies. Amodiaquine, mefloquine, and chloroquine inhibit cellular entry of EBOV in vitro [24–30]. However, conflicting results on the survival impacts with chloroquine treatment in EVD animal models have been reported [24–26, 31–34]. Furthermore, there exists limited clinical evidence for improvements in patient-centered outcomes with the use of empiric antimalarials in EVD care from outbreak settings [13, 23, 35].

During the course of the West Africa EVD outbreak, International Medical Corps (IMC) operated five ETUs, three in Sierra Leone and two in Liberia, with local partners, providing care to over 2,500 patients, including 478 with confirmed EVD. The three ETUs in Sierra Leone received patients who were triaged by a government run District Ebola Response Center, and the two ETUs in Liberia received all patients in their respective geographic areas. All of the ETUs followed IMC guidelines that were developed based on guidance from the WHO and Médecins Sans Frontières collected in previous outbreak settings [36]. Given the frequent use of antimalarial medications in EVD treatment and the limited data on clinical impact, this study evaluated the association between early treatment with the oral antimalarial agent combination artemether-lumefantrine on mortality in patients with EVD using the multinational IMC database.

## Methods

### Study design, setting and population

The data for this retrospective multisite study were derived from five ETUs operated by IMC from 15 September 2014 through 31 December 2015 in Liberia and Sierra Leone [36]. The Sierra Leone Ethics and Scientific Review Committee, the University of Liberia and Rhode Island Hospital Institutional Review Boards provided ethical approval for this study and waived informed consent. Data from those clinical sites were collected during routine patient care and combined into a robust, multi-site database which has previously been employed to assess care provision and patient outcomes in EVD [35–38]. The database was created from the original patient charts by trained staff using standardized clinical records, and data validation and standardization was conducted followed by audits of the data to ensure integrity. Lot quality assurance sampling (LQAS) methods, as previously reported, identified data accuracy at 99% consistency [36]. All patients admitted to the ETUs, who had a final diagnosis of EVD and data on oral antimalarial treatment and mortality were eligible for inclusion. Patients were considered as a part of the treatment group if they received oral antimalarial treatment, regardless of whether or not they received IV antimalarials, given that only a small number of patients received IV antimalarials. Patients who died before ETU triage assessments were excluded.

### Clinical procedures

All patients received care from trained practitioners who used standardized guidelines developed by IMC in consultation with local and trans-national bodies for the treatment of EVD (Additional file 1). The guideline-based care recommended artemether-lumefantrine for all patients, 4 tablets by mouth twice a day for 3 days for adults. For children less than 35 kg (kg), dosing for artemether-lumefantrine was weight based (Additional file 1). Patients unable to take oral antimalarials or who were deemed to have severe malaria were treated with IV antimalarials (artemether or artesunate) until oral medications could be tolerated [39]. Malaria rapid diagnostic test (RDT) results were reported as positive, negative, or not performed for all ETU patients. When undertaken, patients were tested for malaria, using the BinaxNow™ RDT which detects antigens from the *Plasmodium* species of Ovale, Vivax, Malariae, and Falciparum [40]. Patients were tested for malaria when the resources were available and when the staff decided an RDT was clinically helpful. EVD testing was carried out using the real-time reverse transcription polymerase chain reaction (RT-PCR) assays. EVD cycle threshold (CT) values (inversely proportional to viral load) were coded as three categories of > 22 (low viral load), ≤ 22 (high viral load), and missing when results were not available [41].

### Statistical analysis

Statistical analyses were completed using R (Version 1.2.1335) statistical software [42]. Descriptive statistics were conducted for the study population with means and standard deviations (SD) or medians with interquartile ranges (IQR) for continuous variables, and frequencies with corresponding percentages calculated for categorical variables as appropriate. The primary study outcome was mortality during ETU care. Due to the potential for survivor bias derived from the high observed early mortality in this patient population [43], early oral antimalarial treatment was chosen as the primary predictor variable. Early empiric treatment with oral antimalarials was defined as the initiation of treatment with artemether-lumefantrine within the first 48 h of ETU admission. Missing data was present only for RDT and CT assay values, as such this was not corrected for as missing data was not random – patients who died earlier in ETU care were more likely to have missing laboratory data. Due to this, correction for the missing data could bias the results, as has been described in other EVD studies [37, 38, 41].

Inferential analyses were conducted comparing characteristics between the treated and untreated oral antimalarials groups, and between patients that lived and died. Pearson chi-square tests or Fischer’s exact test were used for categorical variables and student’s t-tests or rank-sum tests were used for continuous variables as appropriate. Multivariable logistic regression models for the outcome of mortality during ETU care were constructed to generate adjusted odds ratios (aORs) with associated 95% confidence intervals (CI). Variable selection for inclusion in the multivariable modeling were based on identified evidence of difference (p < 0.05) in the bivariate analyses as well as on previous literature demonstrating associations with mortality in EVD. The variables included in the multivariable models, based on past literature, included: multivitamin (MVI) treatment [44], Cefixime treatment [45], clinical signs and symptoms of bleeding, diarrhea, dyspnea, and dysphagia [7], initial CT value and age [43]. Age was modeled with a cubic spline due to the previously documented quadratic relationship between age and mortality in EVD [43]. As treatments with MVI, Vitamin C and Vitamin A, were found to be colinear in the data, MVI treatment, which included both Vitamin A and C, was utilized in the analytical models. Malaria RDT results were included in the analytical models given their direct relation to the treatment exposure of interest. Country of treatment and oral rehydration solution (ORS) were also included in the models..

Sensitivity analyses, using both stratified and alternative predictor approaches, were completed using adjusted regression modeling as in the primary analysis. This approach has been used in other similar research [37, 38]. One sensitivity analysis assessed the effect of artemether-lumefantrine within the first 48 h of ETU admission stratified based on malaria RDT categorization of negative or unknown. Another analysis stratified the models based on the three possible CT value results, and a final sensitivity analysis evaluated the use of intravenous (IV) antimalarial medications alone or in addition to oral treatment as compared to oral antimalarial medications alone. Due to an identified significant correlation between oral antimalarial administration and paracetamol an additional model incorporating paracetamol as an alternative predictor was performed.

## Results

**Fig. 1:**
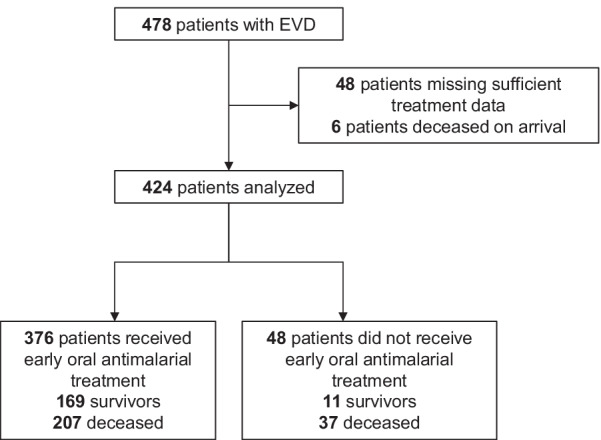
Study flow diagram

From patients treated at the five ETUs, 478 had EVD, and of these 424 were eligible for inclusion (Fig. 1). The average age was 30.5 years (SD ± 18.7 years), and the population was 59.7% female. Overall, the most commonly observed clinical signs and symptoms were diarrhea 298 (70.3%), dysphagia 171 (40.3%), and dyspnea 135 (31.8%). High CT value (low viral loads) were found in 122 (43.4%) of the 281 patients with CT value results, while 159 (56.6%) had low CT values (high viral load). Malaria RDT assessments were carried out on 243 (57.3%) patients, with 48 (19.8%) having a positive result (Table 1).

**Table 1:** Cohort characteristics

	**n (%)**
Age (years)^*^	30.5 (± 18.7)
Sex	
Female	253 (59.7)
Male	171 (40.3)
Length of stay (days)^†^	7 (4, 12)
Country of treatment	
Sierra Leone	290 (68.4)
Liberia	134 (31.6)
Rapid diagnostic test for malaria results	
Positive	48 (11.3)
Negative	195 (46.0)
Not Tested	181 (42.7)
Cycle threshold value /viral load	
≥22 (Low Viral Load)	122 (28.8)
< 22 (High Viral Load)	159 (37.5)
Missing	143 (33.7)
Treatments received in the first 48 hours of care	
Artemether-lumefantrine	376 (88.7)
ORS	373 (88.0)
Cefixime	360 (84.9)
Multivitamins	261 (61.6)
Ondansetron	111 (26.2)
Vitamin C	383 (90.3)
Zinc Sulphate	22 (5.2)
Paracetamol	399 (94.1)
Vitamin A	330 (77.8)
Signs & symptoms in the first 48 hours of care	
Abnormal Bleeding	122 (28.8)
Coma	6 (1.4)
Diarrhea	298 (70.3)
Dysphagia	171 (40.3)
Dyspnea	135 (31.8)
Jaundice	25 (5.9)
Anorexia	283 (66.7)
Abdominal Pain	265 (62.5)
Vomiting	254 (59.9)

*Values represent mean with standard deviation

^†^Values represent median with interquartile range

### Antimalarial treatments

A total of 391 (92.2%) of the overall 424 patients studied received antimalarial treatment at any time point during care. Of those, 11 (2.6%) patients received only IV antimalarials while 31 (7.9%) were treated with both oral and IV antimalarial medications. Early oral antimalarials initiated within the first 48 h of care were provided to 376 cases (88.7%). Evidence of differences were observed between those treated and those not treated with early oral antimalarials based on the country of treatment, receipt of ORS (p < 0.001), cefixime (p < 0.001), multivitamins (p < 0.001), vitamin C (p < 0.001), and vitamin A (p < 0.001); and those with abnormal bleeding (p = 0.001) as compared to those without (Table 2). There was no evidence of oral antimalarial treatment difference among those with different Malaria RDT values or CT values, compared between all groups in each category.

**Table 2:** Cohort characteristics stratified by early oral antimalarial treatment exposure

	Treated (n = 376, %)	Untreated (n = 48, %)	P-value
Age (years)^*^	31.2 ± 18.8	23.0 ± 15.9	0.001
Sex			
Female	223 (59.3)	30 (62.5)	0.756
Male	153 (40.7)	18 (37.5)	
Length of Stay (days)^†^	7, (4, 12)	5, (1, 8)	0.216
Country of Treatment			
Sierra Leone	249 (66.2)	41 (85.4)	0.008
Liberia	127 (33.8)	7 (14.6)	
Rapid diagnostic test for malaria results			
Positive	45 (12.0)	3 (6.3)	0.177
Negative	167 (44.4)	28 (58.3)	
Not Tested	164 (43.6)	17 (35.4)	
Cycle threshold value /viral load			
≥22 (Low Viral Load)	112 (29.8)	10 (20.8)	0.162
< 22 (High Viral Load)	135 (35.9)	24 (50.0)	
Missing	129 (34.3)	14 (29.2)	
Treatments received first 48 hours of care			
ORS	350 (93.1)	23 (47.9)	0.000
Cefixime	342 (91.0)	18 (37.5)	0.000
Multivitamins	243 (64.6)	18 (37.5)	0.000
Ondansetron	97 (25.8)	14 (29.2)	0.604
Vitamin C	357 (94.9)	26 (54.2)	0.000
Zinc Sulphate	19 (5.1)	3 (6.3)	0.727
Vitamin A	315 (83.8)	15 (31.3)	0.000
Signs & symptoms first 48 hours of care			
Abnormal Bleeding	98 (26.1)	24 (50.0)	0.001
Coma	2 (0.5)	4 (8.3)	0.002
Diarrhea	266 (70.7)	32 (66.7)	0.615
Dysphagia	152 (40.4)	19 (39.6)	1.00
Dyspnea	117 (31.1)	18 (37.5)	0.753
Jaundice	22 (5.9)	3 (6.3)	0.754
Anorexia	252 (67.0)	31 (64.6)	0.747
Abdominal Pain	237 (63.0)	28 (58.3)	0.530
Vomiting	223 (59.3)	31 (64.6)	0.534

*Values represent mean with standard deviation

^†^Values represent median with interquartile range

### Mortality outcomes

**Fig. 2:**
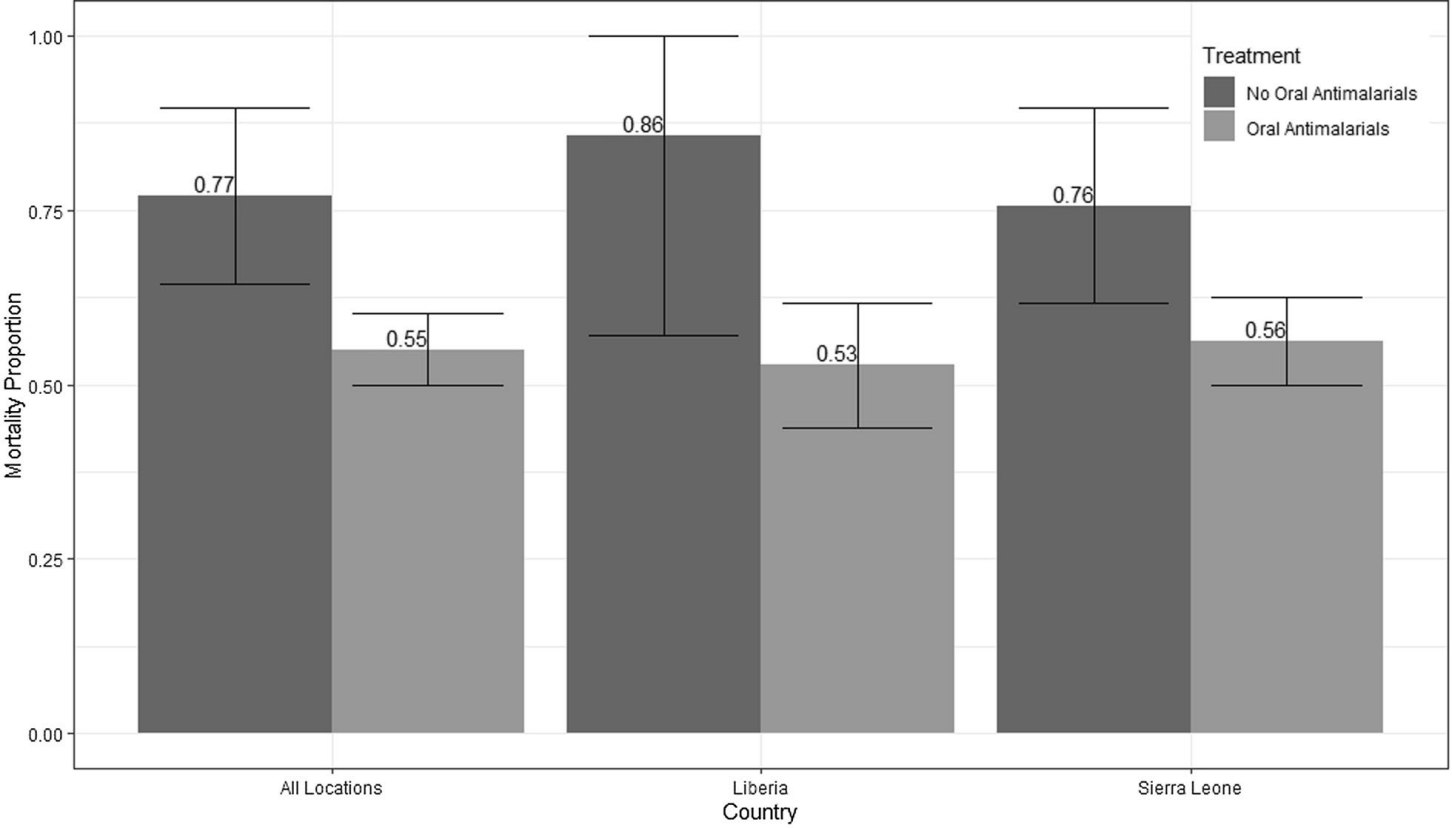
Proportional mortality outcomes by country with 95% confidence intervals

Of the 424 patients included in this analysis, 244 (57.5%) died during treatment, including 207 (55.1%) of the 376 that received oral antimalarials in the first 48 h of care and 37 (77.1%) of the 48 that did not receive oral antimalarials in the first 48 h of care. Across the five ETU sites in Sierra Leone and Liberia, 290 patients were treated in Sierra Leone and 134 were treated in Liberia. Of the patients in Sierra Leone, 171 (59.0%) died during treatment, and of those in Liberia, 73 (54.5%) died during treatment (Fig. 2). There was no evidence of mortality difference between the two countries (p = 0.399, Table 3).

**Table 3:** Cohort characteristics stratified by mortality outcomes

	Survived (n = 180, %)	Died (n = 244, %)	p-value
Age (years)*	28.7 ± 15.3	31.8 ± 20.8	0.080
Sex			
Female	108 (60.0)	145 (59.4)	0.921
Male	72 (40.0)	99 (40.6)	
Length of Stay (days)^†^	13, (10, 17)	4, (3, 6)	0.000
Country of Treatment			
Sierra Leone	119 (66.1)	171 (70.1)	0.399
Liberia	61 (33.9)	73 (29.9)	
Rapid diagnostic test for malaria results			
Positive	17 (9.4)	31 (12.7)	0.082
Negative	94 (52.2)	101 (41.4)	
Not Tested	69 (38.3)	112 (45.9)	
Cycle Threshold Value /Viral Load			
≥22 (Low Viral Load)	81 (45.0)	41 (16.8)	0.000
< 22 (High Viral Load)	50 (27.8)	109 (44.7)	
Missing	49 (27.2)	94 (38.5)	
Treatments received first 48 hours of care			
Artemether-lumefantrine	169 (93.9)	207 (84.8)	0.005
ORS	166 (92.2)	207 (84.8)	0.023
Cefixime	163 (90.6)	197 (80.7)	0.006
Multivitamins	121 (67.2)	140 (57.4)	0.044
Ondansetron	48 (26.7)	63 (25.8)	0.911
Vitamin C	164 (92.8)	219 (89.8)	0.740
Zinc Sulphate	7 (3.9)	15 (6.1)	0.378
Vitamin A	152 (84.4)	178 (73.0)	0.006
Signs & symptoms first 48 hours of care			
Abnormal Bleeding	42 (23.3)	80 (32.8)	0.039
Coma	0 (0.0)	6 (2.5)	0.041
Diarrhea	111 (61.7)	187 (76.6)	0.001
Dysphagia	60 (33.3)	111 (45.5)	0.012
Dyspnea	41 (22.8)	94 (38.5)	0.001
Jaundice	2 (1.1)	23 (9.4)	0.000
Anorexia	116 (64.4)	167 (68.4)	0.405
Abdominal Pain	108 (60.0)	157 (64.3)	0.363
Vomiting	104 (57.8)	150 (61.5)	0.483

*Values represent mean with standard deviation

^†^ Values represent median with interquartile range

Having a high CT value (low viral load) was associated with decreased mortality as compared with having a low CT value or missing CT value. Overall, there were no evidence of differences in Malaria RDT status among those who died as compared to those that lived. Treatment with ORS, cefixime, multivitamins and Vitamin A all had evidence of association with decreased mortality, and increased mortality was associated with patients having signs and symptoms of abnormal bleeding, coma, diarrhea dysphagia, dyspnea, and jaundice (Table 3). In multivariable regression modeling, adjusting for the covariates of age, CT value, bleeding, diarrhea, dyspnea, dysphagia, country of treatment, Malaria RDT status, treatments with cefixime, ORS, and MVI, oral antimalarial treatment provided within the first 48 h of care was associated with reduced mortality (aOR = 0.34, 95% CI: 0.12, 0.92; p = 0.039).

### Sensitivity analysis

Among patients with a negative malaria RDT result, there was no evidence of an association identified with mortality for patients receiving early oral antimalarial treatment (aOR = 0.57, 95% CI: 0.16, 2.0, p = 0.385; Additional file 2). In the analysis of cases in which RDT were not performed, evidence of an association with reduced mortality was observed (aOR = 0.05, 95% CI: 0.00, 0.60, p = 0.034). In stratified sensitivity analyses based on CT value results, there was no observed reduction in mortality across the three categories (low viral loads, high viral load or missing). In assessing the impact of IV treatment, as compared to oral antimalarial treatment alone, no association was identified with mortality (aOR = 0.55, 95% CI: 0.19, 1.53, p = 0.256). Running the multivariate model with the addition of paracetamol as a predictor there was no observed association with mortality outcomes (aOR = 0.79, 95% CI: 0.21, 2.84, p = 0.726).

## Discussion

Although there now exist effective, targeted therapeutics for use in EVD [41], supportive care and utilization of non-EVD specific antimicrobials is recommended in clinical management. However, many of these recommendations, including empirical treatment for malaria infection in EVD outbreaks, are based on limited patient-centered evidence. The data from the current multisite analysis demonstrates that when controlling for confounding factors, there is evidence that treatment with early oral antimalarials was associated with a decrease in mortality. These findings support current treatment guidelines which call for empiric antimalarial medications provided to patients being cared for in EVD outbreak settings.

There is only minimal and conflicting existing data on the impact of antimalarial agents on patient-centered outcomes in EVD [13]. A serologic analysis from a single ETU in Liberia found that plasmodium species parasitemia was associated with increased survival in EVD cases independent of treatment with antimalarial exposure [46]. Gignoux et al*.* found that among EVD-positive patients, the adjusted risk of death was reduced in patients treated with artesunate-amodiaquine versus artemether-lumefantrine [13]. This effect was strongest in EVD-positive patients without malaria co-infection [13]. The authors suggest that this effect may be explained either by activity of amodiaquine against the EBOV, as has been shown in vitro, or another confounding effect [13, 27, 29]. Garbern et al*.* found that, in analyzing data from a population that received community-level mass drug administrations of antimalarial treatments in West Africa, there was a no evidence of change in mortality among patients treated with artesunate-amodiaquine [35]. However, the findings from the current data support the hypothesis that antimalarials confer a beneficial effect in patients with EVD. Furthermore, in the sensitivity analysis, evidence of a protective association was found among patients lacking RDT results and a lack of protective association was noted using the same predictive model with paracetamol, a medication which had statistically significant association with oral antimalarial administration. Insignificant protective effects were noted across sensitivity analyses stratified by all CT value results as well as early in those receiving oral antimalarial treatment with artemether-lumefantrine in the subgroup of patients with a negative malaria RDT result in which the point estimates were in favor of mortality benefit with early oral antimalarial treatment. The lack of evidence in the sensitivity analysis may be due to a type II error derived from the smaller sample sizes in the stratified analyses or could be due to statistical chance. Although there was evidence of an association between a reduction in mortality and early oral anti-malarial treatment, the sensitivity analysis did not yield evidence of this association in RDT negative patients and the literature base is conflicting. The current results should be reinforced by additional prospective studies, drawn from EBV outbreak settings applying standardized international guidelines, for external validation evaluating treatment with artemether-lumefantrine among cases with and without malarial disease in EVD outbreak response care.

A study using computer modeling by Ekins et al*.* found that the antimalarials chloroquine and amodiaquine had favorable antimalarial binding to a protein site likely in EBOV and other filoviruses [47]. Similarly, Goyal et al. demonstrated in vitro inhibition of EBOV by chloroquine and amodiaquine [48]. Although research on artemether-lumefantrine does not specifically exist, the laboratory data indicate that there is a potential biologic activity for EBOV treatment with antimalarial drugs. Given the limited data on the patient-centered impacts of other antimalarial medications, further studies of not only artemether-lumefantrine but other commonly available malaria treatment regimens would be beneficial to inform this area of research.

IV administration of antimalarial medications are the preferred route when patients are experiencing severe illness due to malaria [49]. In severe malaria, gastrointestinal intolerance and erratic intestinal absorption can occur, which may make the use of oral medications unreliable or ineffective [50]. This can be further compounded by coinfection with EVD, where large-volume gastrointestinal fluid loss can occur [49]. Although the regression analysis evaluation of the addition of IV antimalarial treatment found no evidence of impact on mortality, the number of subjects receiving IV treatment were small and those receiving IV medications were likely to have more severe illness, which may not have been adequately controlled for in the modeling approach. As such, definitive understanding of the role of IV antimalarial treatments cannot be concluded from the available data and this treatment approach warrants further study.

### Limitations and strengths

The study has limitations that must be taken into consideration. Treatment with oral antimalarials was not administered in a random approach but rather when the resources were available, potentially allowing for selection bias. This was controlled for as best as possible within the scope of this data with regression analyses, but residual impacts from unidentified confounders are still possible. Additionally, the data did not allow for evaluation of dose–response effects and treatment durations which could be important factors in patient outcomes that warrant further study. All of the patients in this study were treated in the context of IMC operated ETUs, leading to both a standardization of care and data collection, but potentially a decrease in the generalizability of the findings. Country of treatment was controlled for, but, given the limitations of the data, it was not possible to have more granular location control. However, the protocols used at the clinical sites were based on international guidelines and as such the data are likely generalizable to outbreak populations in similar environments where care is generally informed by international recommendations.

One of the strengths of this study is that it is able to draw on validated patient data from a robust database where patient care was standardized across all ETUs. In this, we would like to acknowledge the diligent work done by those who collected this data across all of the ETUs. This study is also able to provide insight into an area where it is not feasible to conduct a randomized control trial to assess outcomes.

## Conclusion

This current study found that artemether-lumefantrine administered early in ETU care in an outbreak setting was associated with decreased mortality in patients with EVD. These data support the empiric provision of antimalarial medications, as currently recommended by international guidelines, however additional prospective studies, in epidemic settings following international guidelines, would be beneficial to validate these findings and enhance our understanding of the impacts of other routes of administration and alternative antimalarial treatment regimes.

## Supplementary Information

**Additional file 1.** International medical corps clinical management guidelines.

**Additional file 2. **Sensitivity analyses for mortality outcomes.

## Data Availability

Data can be made available upon request from the corresponding author.

## Abbreviations

EVD: Ebola virus disease
ETUs: Ebola Treatment Units
WHO: World Health Organization
EBOV: Ebola Virus
IMC: International Medical Corps
RDT: Rapid diagnostic test
RT-PCR: Reverse transcription polymerase chain reaction
CT: Cycle threshold
IQR: Interquartile ranges
aORs: Adjusted odds ratios
CI: Confidence intervals
MVI: Multivitamin
ORS: Oral rehydration solution
IV: Intravenous
