# Supplementary material for: Association between oral antimalarial medication administration and mortality among patients with Ebola virus disease: a multisite cohort study
Source: BMC Infect Dis. 2022 Jan 20;22:71. doi: 10.1186/s12879-021-06811-3 (PMC8772178; doi:10.1186/s12879-021-06811-3)
Supplement: Supplementary file 1 — Additional file 1. International medical corps clinical management guidelines. [file 12879_2021_6811_MOESM1_ESM.pdf]

Association between Oral Antimalarial Medication Administration and Mortality Among Patients with Ebola Virus Disease: A Multisite Cohort Study

Logan Abel, Shiromi M. Perera, Derrick Yam, Stephanie Garbern, Stephen B. Kennedy, Moses Massaquoi, Foday Sahr, Dayan Woldemichael, Tao Liu, Adam C. Levine, Adam R. Aluisio

*This document provides a general overview of procedures, which varied slightly by ETU and over time.

Additional file 2: International Medical Corps Viral Hemorrhagic Fever Guidelines

International Medical Corps

Viral Hemorrhagic Fever
Draft Guidelines

Standard Clinical Procedures for Ebola Treatment Unit (ETU) Operations*

Version 1.0, April 13, 2016

ETU Triage Algorithm for Suspect Cases

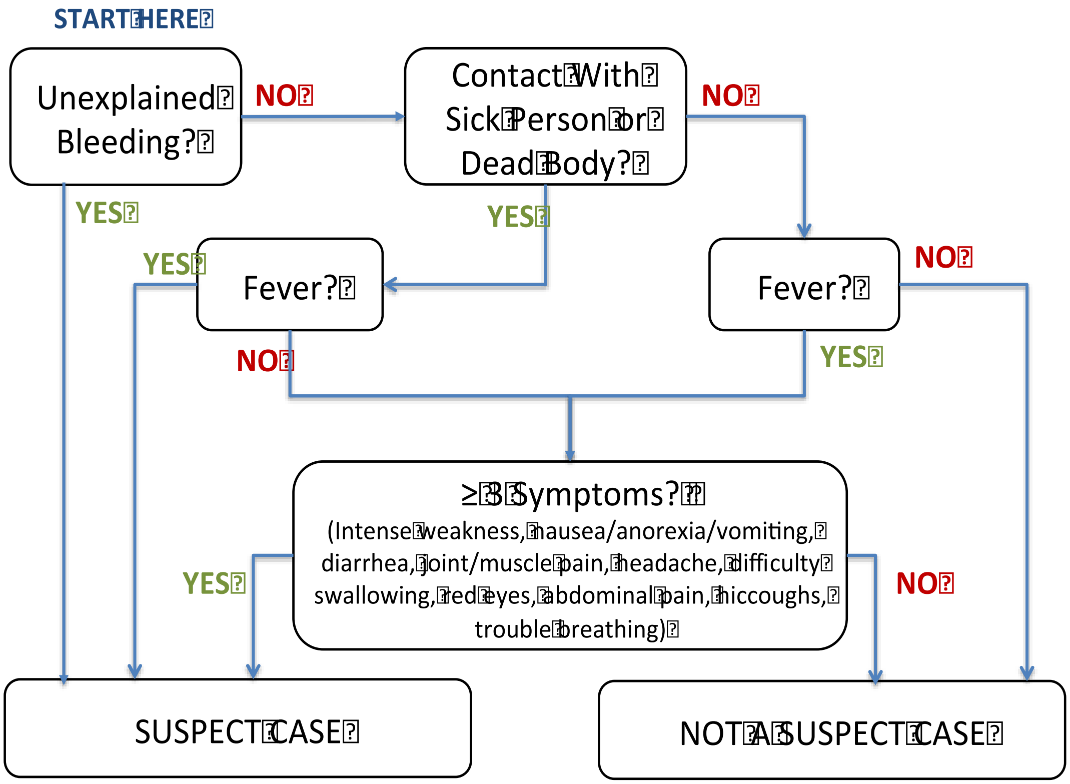
START HERE
Unexplained Bleeding?
NO
Contact With Sick Person or Dead Body?
NO
YES
YES
Fever?
YES
NO
Fever?
NO
YES
≥3 Symptoms?
(Intense weakness, nausea/anorexia/vomiting, diarrhea, joint/muscle pain, headache, difficulty swallowing, red eyes, abdominal pain, hiccoughs, trouble breathing)
YES
NO
SUSPECT CASE
NOT A SUSPECT CASE

Standard Clinical Procedures

<u>Admission</u>

1. An admission form/epidemiological tracing form will be filled out by the triage nurse for all patients on arrival:
    a. Patient meets the triage criteria for a suspect case (see triage criteria) and will be admitted to the suspect ward
    b. Patient had a positive test for Ebola in the community or another facility and will be admitted to the confirmed ward
    c. Patient is dead on arrival to the ETU but meets the triage criteria for a suspect case or had a positive Ebola test in the community or at another facility and will be moved to the morgue

2. After filling in the admission/epidemiologic form, the patient's name and ID should be written on a bracelet by the triage nurse.

3. Next the triage nurse should fill in a lab slip for the patient and write their name and ID on an EDTA tube.

4. The triage nurse should explain to the patient that they are being admitted to the ETU and what to expect once they are admitted.

5. Two hygienists in PPE should place the bracelet on the patient's wrist and then escort (or carry) the patient to the suspect (or confirmed) ward. The triage nurse should hand over the bracelet, lab slip, and EDTA tube for the hygienists to take with the patient.

6. Afterwards, disinfection of the area where the patient sat or came into contact with should be sprayed with 0.5% chlorine solution.

<u>Daily Rounds</u>

1. A physician/physician assistant will round on all patients in the ETU at least once a day. During their rounds they will:
    a. Assess the patient clinically
    b. Fill in the daily symptom log
    c. Write orders for medications/labs as needed

2. Nurses will round on all patients at least 4 times per day (morning, mid-day, evening, and night). During rounds they will:
    a. Check weight (for children) on their first day
    b. Check patient temperature, pulse, and respirations once per day
    c. Give patients oral and intravenous medications and fluids
    d. Perform lab testing as ordered

3. Nurse assistants will round on all patients at least 4 times per day (morning, mid-day, evening, and night). During rounds they will:
    a. Deliver food to patients and encourage them to eat
    b. Give water/ORS to patients and encourage them to drink
    c. Clean and help change patients as needed

4. Psychosocial (PSS) will round on all patients at least once per day. During rounds they will:
    a. Check on patients well-being, comfort and needs
    b. Provide basic PSS support in line with principles of PFA
    c. Ensure connections and communication with family

<u>Laboratory Testing</u>

1. All patients should have an intravenous catheter placed and their blood drawn for Ebola testing on arrival in an EDTA (purple top) tube.
2. Initial testing should also include PCR for malaria.
3. Result of initial lab test will determine whether the patient remains in suspect/probable ward or moved to confirmed ward.
4. If initial test negative patient will remain in ETU and have further PCR test to ensure 2 negative tests prior to discharge.
5. Patients who receive initial positive test will be moved to the confirmed ward, further PCR testing will only take place after patient becomes symptom free.
6. All patients who die during admission to the suspect and probable ward or who are dead upon arrival to the facility will have a buccal swab taken for PCR testing prior to being moved to the morgue.

<u>Medications</u>

1. All patients who are able to drink should be given premixed Oral Rehydration Solution (ORS) and encouraged to drink it all each day:
    a. Adults: 1.5 liters per day of ORS by mouth
    b. Children: 40cc/kg per day of ORS by mouth
    c. Adults and children with diarrhea and dehydration should be given additional ORS – see dehydration protocol

2. All patients should be given a broad spectrum antibiotic for 5 days:
    a. Adults: cefixime 400mg tablet by mouth once daily for 5 days
    b. Children: cefixime 8mg/kg solution by mouth once daily for 5 days
    c. Alternatively, ciprofloxacin 500mg twice daily for patients > 7 years and amoxicillin 20mg/kg twice daily for patients < 7 years

3. All patients should be given Coartem for 3 days:
    a. Adults (>35kg): 4 tablets by mouth twice daily for 3 days
    b. Children (25-34kg): 3 tablets by mouth twice daily for 3 days

c. Children (15-24kg): 2 tablets by mouth twice daily for 3 days
d. Children (5-14kg): 1 tablet by mouth twice daily for 3 days
e. Coartem can be crushed and mixed in water for children

4. All patients should be given paracetamol for pain/fever:
    a. Adults: 1 gram by mouth 4 times per day (as needed)
    b. Children: 15mg/kg by mouth 4 times per day (as needed)

5. All patients should be given omeprazole to prevent ulcers/gastritis:
    a. Adults + Children > 20kg: omeprazole 20mg by mouth once daily
    b. Children < 20kg: omeprazole 10mg (half capsule) by mouth once daily (mix in water)

6. All patients should be given vitamin A supplementation:
    a. Adults: 200,000 IU by mouth once daily on days 1, 2, 8
    b. Children 6-12mo: 100,000 IU by mouth once daily on days 1, 2, 8

7. All patients should be given vitamin C supplementation:
    a. Adults: 500mg by mouth 3 times per day
    b. Children: 250mg by mouth 3 times per day (crush and mix in water)

Symptom Specific Protocols

Pain

1. Pain is a common symptom of Ebola Virus Disease (EVD), including headache, sore throat, neck pain, back pain, chest pain, abdominal pain, joint pain, and muscle pain.

2. Treating pain of both adults and children is an important part of care for patients in the ETU.

3. All patients in the ETUETU will be given paracetamol for pain as part of their standing orders:
    a. Adults: 1 gram by mouth 4 times per day (as needed)
    b. Children: 15mg/kg by mouth 4 times per day (as needed)

4. Patients who require stronger medicine for pain can be given oral morphine:
    a. Adults: morphine sulfate 10mg by mouth 4 times per day as needed (can be increased to 20mg or even 30mg if needed for pain)
    b. Children: morphine sulfate 0.3mg/kg by mouth 4 times per day as needed for pain (can be increased to 0.5mg/kg)

5. Patients who are unable to swallow tablets can receive morphine by IV:
    a. Morphine hydrochloride 0.1mg/kg IV four times per day as needed for both adults and children
    b. If the patient does not have an IV in place, then morphine hydrochloride can be given sub-cutaneous (SC) at the same dosage
    c. Avoid intramuscular (IM) injection due to risk of bleeding into muscle and formation of hematoma

6. Patients should not be given aspirin, ibuprofen, or other NSAIDs due to risk of bleeding.

Nausea/Vomiting

1. We should treat nausea early in order to prevent patients from vomiting and also to help them eat and drink.

2. For mild nausea, we can give metoclopramide by mouth:
    a. Adults: metoclopramide 10mg by mouth every 6 hours for nausea
    b. Children: metoclopramide 0.1mg/kg by mouth every 6 hours for nausea

3. For patients with vomiting who cannot take oral metoclopramide, we can give metoclopramide by IV:
    a. Adults: metoclopramide 10mg IV every 6 hours for vomiting
    b. Children: metoclopramide 0.1mg/kg IV every 6 hours for vomiting

<u>Diarrhea/Dehydration</u>

1. Many of the patients who die of EVD actually die from dehydration due to diarrhea. We can prevent these deaths by rehydrating patients appropriately.

2. All patients with diarrhea should be assessed for signs of dehydration:
    a. Severe Dehydration (2 or more signs):
        i. Lethargic or unconscious
        ii. Not able to drink
        iii. Sunken eyes
        iv. Very slow skin pinch
    b. Some Dehydration (2 or more signs):
        i. Restless or irritable
        ii. Drinking eagerly
        iii. Sunken eyes
        iv. Slow skin pinch

3. Patients with severe dehydration should receive intravenous fluids (see Intravenous (IV) Catheter Protocol below):
    a. Adults
        i. First give Ringers Lactate 2 liters over 1 hour
        ii. Reassess patient during the next rounds
        iii. If patient still has severe dehydration, given an additional 2 liters of Ringers Lactate over 1 hour
        iv. Continue this process until patient no longer has severe dehydration
        v. Encourage patient to begin drinking ORS as soon as they are able to do so
    b. Children
        i. First give Ringers Lactate 30cc/kg over one hour
        ii. Reassess patient during the next rounds for signs of severe dehydration as well as trouble breathing
        iii. If patient still has severe dehydration but no trouble breathing, given an additional 30cc/kg of Ringers Lactate over 1 hour
        iv. Repeat this process until the severe dehydration has resolved
        v. Begin providing ORS as soon as child is able to start taking sips

4. Patients with some dehydration should be given extra oral rehydration solution and encouraged to drink it all within 4 hours by taking slow sips.
    a. Adults: 3 liters of ORS by mouth over 4 hours
    b. Children 5-14 years: 1.5 liters of ORS by mouth over 4 hours
    c. Children < 5 years: 50ml/kg of ORS by mouth over 4 hours

5. **Giving ORS does not mean just putting the bottle of ORS by the patient!**
    a. Adult patients should be encouraged to drink small sips every few minutes until the bottle is finished.
    b. Children should have a caregiver who can give them small sips using the cap of the ORS bottle.
    c. Patients who are too weak to lift the bottle should be given a straw made from a piece of IV tubing to drink the ORS.

6. Give zinc to children under five years with diarrhea
    a. > 6 months: zinc sulfate 20mg tab once per day for 10 days
    b. < 6 months: zinc sulfate 10mg tab once per day for 10 days
    c. Zinc can be mixed in water and given to child

<u>Hemorrhage</u>

1. Many patients with Ebola will have some form of bleeding. The most common sites of bleeding will be blood in the stool, bleeding from the vagina, bleeding from the gums, bleeding from the nose, vomiting blood, and bleeding from an IV catheter site.

2. For bleeding from an IV catheter site:
    a. If the IV is no longer needed, the IV should be removed and the site should be bandaged well to stop bleeding.
    b. If the IV is still needed and there is only a little bleeding, wrap additional bandage around the arm to stop the bleeding.
    c. If there is a lot of bleeding from the catheter site, remove the IV and bandage the site well to stop bleeding.

3. For bleeding from the nose, instruct the patient to pinch just below the bridge of their nose tightly for 30 minutes at least.

4. For bleeding from the gums, instruct the patient to rinse their mouth with clean water several times a day and spit out into their bucket.

5. For vomiting blood, give metoclopramide as instructed in the nausea/vomiting section above.

6. For blood in the stool, double the patient’s current dose of omeprazole from 20mg to 40mg for an adult and from 10mg to 20mg for a child.

Anxiety/Confusion

1. Many patients with Ebola will have anxiety or confusion at some point during their stay in the ETU. It is important to treat symptoms of anxiety or confusion **early** before they become more severe and difficult to manage.

2. If patients are experiencing anxiety or confusion, they should be provided basic PSS support including listening supportively to their concerns, providing reassurance and meeting unmet needs. The PSS team should also be consulted and can provide additional support.

3. Patients with moderate to severe anxiety who are not sufficiently responding to basic PSS support and are able to swallow pills should be given diazepam:
    a. Adults: diazepam 5mg by mouth up three times a day as needed.

4. Patients with moderate to severe confusion or agitation who are not sufficiently responding to basic PSS support can be given chlorpromazine:
    a. Adults: chlorpromazine 50mg by mouth three times daily as needed.

5. Patients with severe confusion or agitation should be treated with an IV medicine if they have an IV in place already and it can be done safely:
    a. Adults: haloperidol 5mg IV or lorazepam 1mg IV as needed
    b. Children: lorazepam 0.05mg/kg IV as needed

6. **You should never try to place a new IV or give an injection in a patient with confusion or agitation.**

7. If absolutely needed for a patient's safety, soft restraints (i.e. bed sheets) can be used to tie a patient to their bed to keep them from falling off.
    a. If soft restraints are being placed, at least two limbs should be restrained (1 arm and 1 leg, or 2 arms, or 2 legs).
    b. Patients need to be monitored consistently while restrained
    c. The restraints should be removed as soon as it is safe to do so.
    d. PSS support should be provided before, during and after an episode of severe confusion or agitation

Intravenous Catheter Protocol

IV lines in Ebola case patients can be done, provided that the benefits outweigh the risks both to the patients and staff. IV lines should be inserted by an appropriately trained member of the medical team.

At admission, all patients will get an intravenous (IV) line to be inserted by trained medical staff. During insertion, a blood sample for lab analysis (PCR) will be taken.

Some patients may require IV rehydration to restore fluid volume or correct electrolyte imbalances. (Note: children and elderly patients with Ebola are more likely to develop fluid imbalances.) **The decision to give IV fluids is to be made by the doctor or physician's assistant.** To prevent potentially life-threatening hypovolemic shock, early recognition of patients who are not able to take oral rehydration fluids is paramount.

**Flushing and Blood Draws:**	
Infusions	Blood draws
- Use 3 ml water for injection, infuse medication/fluids, flush with 3 ml water after injection<br>- Follow doctor's orders on all IV fluids and medications	- Draw 1-2 ml of blood for lab analysis (PCR) at the insertion point.<br>- Ensure use of correct sample bottles and correct labeling of these bottles
**Important Notes:**	
- For insertion, at least 2 staff wearing complete PPE are required<br>-Safety box and disposing bin should be directly next to staff during this time<br>- Do not insert in uncooperative or violent patients<br>- Do not insert in patients who have signs and symptoms of internal/external hemorrhage<br>- If you miss the first insertion attempt, consult another doctor or nurse<br>**- Do not insert if you feel unsafe; your safety comes first**	

Nutrition Protocols

The following protocols are to guide ETU staff in administration of proper nutrition to patients affected with EVD.

Supply and distribution of food

1. All patients will be provided with three meals per day consisting of healthy local food, consistent with a typical diet. This will be prepared and delivered to the hospital.
2. Food will be distributed during morning, midday, and night rounds by staff members wearing appropriate PPE.
3. If food is provided by the family, it can be distributed during the appropriate round, but family members are not allowed into the treatment ward to distribute food.

Assisting with feeding

1. If a patient cannot eat on their own, staff will assist to the best of their ability to administer the food.
2. If there are children who cannot feed themselves, they will be assisted either by staff, a parent, or by another adult in the unit who has taken on the role of caregiver.
3. Any patient who cannot swallow due to throat pain or weakness will be given plumpy'nut and encouraged to eat as much as possible. They will also be given juice or any food which with they feel comfortable.
4. Meals may be tailored to meet the needs of patients.

Supplements and medication administration to assist with nutrition

1. All patients will be provided with Vitamin A and C supplementation as per medication protocols.
2. Patients who cannot eat due to severe vomiting and nausea will be provided with anti-emetics pre meals as per medication orders and encouraged to eat by staff.

Breastfeeding and child health

1. If a breastfeeding mother or baby are admitted to the ward as suspected cases of EVD, breastfeeding should immediately cease and alternative nutrition should be provided.
2. Any child who was previously breastfed should be given a breast milk substitute up to 24 months.
3. Formula should always be prepared appropriately to offer maximum nutrition and all staff should be trained in this preparation.

4. Bottles should not be used and open feeding cups should be cleaned adequately to prevent the spread of infection.

<u>Frequency of feeding</u>

- Aged 0 - 8 weeks: Feed 8-12 times per 24 hours or on demand. If the child is severely ill they may not ask to feed and will have to be encouraged as frequently as possible.
- Aged 2 - 4 months: Babies should be fed on average 6 times per 24 hours.
- Aged 4 - 7 months: Babies should be fed on average 5 times per 24 hours. Complementary feeding should commence at six months of age. This should consist of appropriate, healthy local foods.
- Aged 7 - 12 months: Babies should be fed 4 times per 24 hours.

Wet nurses should be discouraged for babies with mothers who are suspected or confirmed Ebola.

Psychosocial Support in ETUs – Protocol Overview

The aim of psychosocial (PSS) support is to promote and protect the psychological wellbeing of all patients and their families. PSS support in ETUs is targeted at:

- Patients - as part of providing compassionate care to reduce fear and suffering and to ensure patient dignity is preserved.
- Their families - to ensure provision of adequate understanding of the patient's clinical condition with the goal of reducing the impact of stress, fear, and stigma. In addition, to support their current emotional state and to help them better understand the processes of the patient's illness, hospitalization course, death, and burial.
- Healthcare workers - to support positive ways of coping with stressors brought about from caring for very sick and highly infectious individuals and often being faced with stigma and isolation from families and communities.
- The community - to facilitate understanding of EVD including transmission, prevention and response, encourage acceptance of outbreak control measures, and reduce the social isolation of patients, their families, survivors, and healthcare workers.

1. Initial Contact

<u>IF Patient arrives at the ETU as a walk in with family:</u>
While the patient is triaged and admitted, the allocated PSS Officer introduces themselves to the family in the Family Area. While talking with the family, PSS officers abide by the infection control protocols (e.g. do not touch the family members; only sit on the designated chairs and ensure the family sits on the other designated chairs; Keep a safe distance of 3 feet or one meter).

- The family are given the contact details for the PSS ETU team and encouraged to go home and wait for the surveillance team to visit as they are contacts and need to be monitored for 21 days.
- The aim of this interaction is to relieve distress of the family, orientate them to the site, deliver health promotion messages, and deliver dos and don'ts of the ETU. IF the family want to talk, PSS officers allow them to talk about what happened but do not force them in line with principles of PFA.

<u>IF the patient arrives at the ETU in an ambulance or without family:</u>
The allocated PSS officer liaises with Triage nurses and the nursing team to find out basic details, physical health, risks, and contact details for the individual and their family.

IF an unaccompanied child is admitted to the ETU, the allocated PSS team must inform the designated Representative from the Ministry of Social Welfare and

other relevant agencies (e.g. UNICEF or other Family Tracing and Reunification Partners).

The initial visit between the PSS Support (PSS) team and the patient takes place at the ETC after triage has occurred and the patient is on the suspect ward. Initial contact is made within 1-2 hours of the patient arriving. The aim of the introduction is to relieve distress, offer some calm support, identify yourself, try and understand how the person is feeling and what is happening to them. The initial introduction covers the following:

- Introduction of the PSS team tasks and team member(s)
- Explanation of role of PSS team including daily visits, availability to talk, and keeping contact with family
- Offering to reach out and assisting with keeping connections and communication links between the patient and their family and community
- Asking about needs (e.g. communication with family, specific food or drink)
- Listening supportively without forcing patients to talk in line with principles of PFA.

PSS Support offered to ETU-admitted patients

PSS support to patients and families:

- Each inpatient is assigned a PSS officer who will be responsible for the PSS care of that individual and their family.
- Communication between patients and PSS officers takes place outside (over the designated fence). In rare circumstances and on a case by case basis, PSS officers see patients who are too sick to come outside (wearing PPE).
- PSS officers will have at least one conversation to check in with their patients (and where applicable) family members per day.
- The PSS team also organizes group sessions for patients and families including health promotion messages, psycho-education (e.g. coping with psychological distress, normal stress reactions), group activities (e.g. recreational and social activities) while in the ETU.
- The PSS team organizes access to social and recreational activities and materials for adults and children, including showing movies in the evenings (via projector on a big screen), providing radios, reading materials and toys/games.
- PSS staff promote communication between patients and families (see details below).

Ensuring connection and communication between patients and families:

- With the patient’s consent, PSS officers offer community PSS outreach services to their family and communities and will also establish communication links between the individual and family at the ETU.

- PSS teams contact the patient’s family by mobile phone and/or community outreach visit to engage the family and reduce distress in the communities.
- During family visits, the PSS officer helps the family to walk to the family visiting area so that they can see their loved ones.

Involvement of the PSS team as part of the ETU:

1. PSS staff to attend all medical team handovers every morning and afternoon.
2. PSS officers liaise with medical staff, hygienists, WASH staff to ensure that the dignity of the patient is always at the forefront of staff’s minds and that various patient needs and concerns are addressed to the extent possible.

Discharge

When patients recover, PSS officers work closely to prepare for their eventual ETU discharge.

- PSS officers contact the family and conduct outreach work to ensure that the family and the community are ready to receive them.
- PSS officers contact other relevant authorities or service providers (e.g. Ministry of Social Welfare) to ensure information sharing and follow up with needed services and supports.
- One to two days before discharge, PSS team offer PSS discharge interview to identify patient’s needs and coping strategies and to create a realistic plan as to how the person can cope and what to expect from their community.
- If needed, PSS officers organize transport for the patient for the next day depending on distance required to travel.
- On the day of discharge PSS officers explain what is in the discharge packages from IMC and other organizations.
- At the time of discharge after the patient has washed and changed clothes, a discharge event takes place with dancing, drumming and singing (as the person comes outside) to celebrate person’s future.
- Before, during and after discharge, PSS officers offer health promotion/support messages to the individual and family.

Burial

When patients pass away, the PSS team facilitates family contact, family support to view the body in the morgue, and work closely with burial teams to ensure that the families are satisfied with the way their loved ones were treated in death. The PSS team also facilitates continued communication with the family as needed and visitation of burial sites.
