# Supplementary material for: Association between oral antimalarial medication administration and mortality among patients with Ebola virus disease: a multisite cohort study
Source: BMC Infect Dis. 2022 Jan 20;22:71. doi: 10.1186/s12879-021-06811-3 (PMC8772178; doi:10.1186/s12879-021-06811-3)
Supplement: Supplementary file 2 — Additional file 2. Sensitivity analyses for mortality outcomes. [file 12879_2021_6811_MOESM2_ESM.docx]

**Association between Oral Antimalarial Medication Administration and Mortality Among Patients with Ebola Virus Disease: A Multisite Cohort Study**

Logan Abel, Shiromi M. Perera, Derrick Yam, Stephanie Garbern, Stephen B. Kennedy, Moses Massaquoi, Foday Sahr, Dayan Woldemichael, Tao Liu, Adam C. Levine, Adam R. Aluisio

Additional file 2: Sensitivity Analyses for Mortality Outcomes

| *Mortality Stratified By Malaria Rapid Diagnostic Test Categorization* | | | |
| --- | --- | --- | --- |
| Rapid Diagnostic Test Categorization | aOR^*†^ | 95% Confidence Interval | p-value |
| Negative | 0.57 | 0.16, 2.0 | 0.385 |
| Not Tested | 0.05 | 0.00, 0.60 | 0.034 |
| *Mortality Stratified By Cycle Threshold Value* | | | |
| Cycle Threshold Value | aOR^*‡^ | 95% Confidence Interval | p-value |
| > 22 (Low Viral Load) | 0.65 | 0.14, 3.05 | 0.579 |
| < 22 (High Viral Load) | 0.17 | 0.01, 1.92 | 0.145 |
| Missing | 0.16 | 0.01, 1.23 | 0.119 |

* Abbreviates adjusted Odds Ratio.

† Multivariable models adjusted for: age, cycle threshold value, country of treatment, symptoms of bleeding, diarrhea, dyspnea, dysphagia, treatments with cefixime, oral rehydration solutions and multivitamins

‡ Multivariable models adjusted for: age, country of treatment, malaria Rapid Diagnostic Test characterization, symptoms of bleeding, diarrhea, dyspnea, dysphagia, treatments of cefixime, oral rehydration solutions and multivitamins
